# Measurement of genetic diseases as a cause of mortality in infants receiving whole genome sequencing

**DOI:** 10.1038/s41525-020-00155-8

**Published:** 2020-11-02

**Authors:** Stephen F. Kingsmore, Audrey Henderson, Mallory J. Owen, Michelle M. Clark, Christian Hansen, David Dimmock, Christina D. Chambers, Laura L. Jeliffe-Pawlowski, Charlotte Hobbs

**Affiliations:** 1 Rady Children’s Institute for Genomic Medicine, San Diego, CA 92123 USA; 2 Department of Pediatrics, University of California, San Diego, CA 92123 USA; 3 Department of Epidemiology and Biostatistics, University of California San Francisco, San Francisco, CA USA

**Keywords:** Molecular medicine

## Abstract

Understanding causes of infant mortality shapes public health policy and prioritizes diseases for investments in surveillance, intervention and medical research. Rapid genomic sequencing has created a novel opportunity to decrease infant mortality associated with treatable genetic diseases. Herein, we sought to measure the contribution of genetic diseases to mortality among infants by secondary analysis of babies enrolled in two clinical studies and a systematic literature review. Among 312 infants who had been admitted to an ICU at Rady Children’s Hospital between November 2015 and September 2018 and received rapid genomic sequencing, 30 (10%) died in infancy. Ten (33%) of the infants who died were diagnosed with 11 genetic diseases. The San Diego Study of Outcomes in Mothers and Infants platform identified differences between in-hospital and out-of-hospital causes of infant death. Similarly, in six published studies, 195 (21%) of 918 infant deaths were associated with genetic diseases by genomic sequencing. In 195 infant deaths associated with genetic diseases, locus heterogeneity was 70%. Treatment guidelines existed for 70% of the genetic diseases diagnosed, suggesting that rapid genomic sequencing has substantial potential to decrease infant mortality among infants in ICUs. Further studies are needed in larger, comprehensive, unbiased patient sets to determine the generalizability of these findings.

## Introduction

Infant mortality has been tracked annually worldwide for many years (http://www.geoba.se/population.php?pc=world&type=019&year=2017&st=rank&asde=d&page=1, https://data.worldbank.org/indicator/SP.DYN.IMRT.IN?locations=US). Knowledge of causes of infant mortality shapes public health policy and prioritizes diseases for investments in surveillance, intervention and medical research. As a result of such prioritization, programs such as Back to Sleep/Safe to Sleep® have significantly reduced rates of many causes of infant mortality over the past 50 years in the United States (https://safetosleep.nichd.nih.gov/, https://www.cdc.gov/reproductivehealth/maternalinfanthealth/infantmortality.htm.)^1–4^. However, knowledge of the underlying causes of infant mortality is based on death certificates, which are estimated to be inaccurate or incomplete in 33–53% of cases^5–10^. In addition, the vast majority of death certificates are not informed by molecular diagnoses.

Congenital malformations, deformations and chromosomal abnormalities (International Classification of Diseases (ICD-10) codes Q00–Q99) have been reported to be the leading causes of infant death in the United States for the past 50 years^1–4^. Many, but not all, of these are associated with genetic diseases. Several of the other nine leading causes of infant mortality are also associated with genetic diseases, including low birth weight and prematurity, sudden infant death syndrome, newborn sepsis, diseases of the circulatory system and neonatal hemorrhage^1–4^. Molecular autopsies have transformed the forensic evaluation of sudden unexplained death in the young, leading to new guidelines and treatments and a decreased number of deaths^9,10^. Despite this success, and while the molecular basis of 14,000 genetic diseases has been determined^11,12^, it is not known which genetic diseases are leading contributors to infant mortality other than for sudden unexplained death and chromosomal aneuploidies.

Knowledge of the contribution of genetic diseases to infant mortality has grown indirectly. Many infant deaths occur in hospitals, particularly in neonatal, pediatric and cardiovascular intensive care units (ICUs)^13,14^. Rapid genomic or precision medicine has created a new opportunity to decrease infant mortality associated with genetic diseases in ICUs^15–45^. Many genetic diseases have specific, effective, evidence-based treatments. However, when diagnosis of a genetic disease is delayed or absent, treatments must be chosen empirically, with suboptimal outcomes, including unnecessary infant mortality. In response, rapid and ultra-rapid clinical genomic sequencing methods have been developed for prompt diagnosis in infants and children in ICUs who may have a genetic disorder for which effective treatment exists^15–45^. It is estimated that genetic diseases occur in about 16% of infants in regional ICUs^24,46^. Over the past 5 years, rapid genomic sequencing has demonstrated consistent utility in this population. In 873 children in ICUs described in 13 studies, 36% received a genetic disease diagnosis by rapid genomic sequencing^17,20,21,23–26,28,30,33,34,36,38–45^. The average clinical or therapeutic utility of those diagnoses was 71%. Changes in patient outcomes were reported in 27%. The latter included both avoidance of infant mortality by therapeutic interventions and palliative care decisions. These data suggest that clinical-grade genomic sequencing has the analytic and diagnostic performance to ascertain the contribution of genetic diseases to infant mortality. Furthermore, determination of the leading genetic diseases associated with infant mortality may provide information to direct clinical and public health interventions to reduce infant mortality and decrease suffering. The current manuscript reports results of a scoping study and literature review to assess whether genetic diseases in toto are a leading cause of infant mortality.

## Results

### Cohort Studies

Among 312 probands who had received rapid genomic sequencing in two research datasets, we examined the characteristics of infants who died prior to their first birthday. Both studies enrolled inpatient infants at Rady Children’s Hospital in San Diego between 11/23/2015 and 9/7/2018, primarily from a level IV neonatal intensive care unit. Of these, 205 infants were enrolled in the NSIGHT2 randomized controlled trial^24^ and 107 infants were enrolled under a biorepository protocol^17,18,23,30^. All infants had an acute illness at the time of enrollment, with a disease of unknown, possibly genetic, etiology. At one year follow up, 30 (9.5%) of 312 enrollees had died in infancy. They represented approximately 7% of infant deaths in San Diego County during this period (https://www.sandiegocounty.gov/content/dam/sdc/hhsa/programs/phs/documents/MchSt-InfMort.pdf).

We compared the demographic characteristics of infants who died with those who survived in the two cohorts, and with those of all infant deaths in San Diego County during the same period. Among participants in the two research cohorts, infant mortality did not differ significantly between males (9.5%; 16 of 169) and females (12.3%; 14 of 114; *P* = 0.45). In contrast, in San Diego County from 2015–2019, infant mortality was lower among females (0.35%, 282 of 81,612) than males (0.42%, 359 of 85,710, *p* < 0.02) (https://www.sandiegocounty.gov/content/dam/sdc/hhsa/programs/phs/documents/MchSt-InfMort.pdf). Excluding 23 infants of unknown race, mortality in the two studies did not differ significantly between white infants (10.3%; 19 of 185 infants) and those of other races (9.6%; 10 of 104, *P* = 0.86). In contrast, in San Diego County from 2015–2019, white infants had a lower mortality (0.34%, 199 of 58,224) than non-white infants (0.41%, 444 of 109,100; *p* < 0.05) (https://www.sandiegocounty.gov/content/dam/sdc/hhsa/programs/phs/documents/MchSt-InfMort.pdf). Excluding 8 infants of unknown ethnicity, mortality did not differ significantly between Hispanic infants (11.7%; 16 of 137 infants) and non-Hispanic infants (8.4%; 14 of 167; *P* = 0.34) in the two studies. In San Diego County from 2015–2019, Hispanic infants had a higher mortality rate (0.45%, 304 of 66,928) than non-Hispanic infants (0.34%, 339 of 100,396; *P* < 0.0002) (https://www.sandiegocounty.gov/content/dam/sdc/hhsa/programs/phs/documents/MchSt-InfMort.pdf).

Among the 30 infants in the research studies who died, we evaluated whether the likelihood of death had been anticipated or palliative care discussed with parents by EHR review. Eleven (37%) of 30 deceased infants had received a palliative care consult, and 18 (60%) of 30 deceased infants had a modified “Code Blue” resuscitation status in the event of cardiorespiratory arrest. Of these, 16 (53%) had “allow natural death” as their Code Blue status at time of death and 2 (7%) had a partial resuscitation Code Blue status. Of the infants who had received a genetic diagnosis, 4 (40%) out of 10 had a palliative care consult, and 7 (70%) out of 10 had a modified “Code Blue” resuscitation status. Of the 20 infants who did not receive a genetic diagnosis, 7 (35%) had a palliative care consultation and 11 (55%) had a modified “Code Blue” status.

We examined phenotypic characteristics of the infants who died and survived in the two studies. Using methods that had previously been validated for identification of patients with orphan diseases^17^, we extracted HPO terms corresponding to the clinical features of the 312 infants from unstructured text in the Rady Children’s Hospital EHR at time of enrollment (prior to death) using clinical natural language processing. Of 2,313 HPO terms extracted from Epic only four terms differed in frequency significantly between enrollees who died in infancy and those who survived (Table 1)—abnormal urinary system, respiratory failure, hypotension and cardiac arrest. All occurred in a higher proportion of records of infants who died.

**Table 1:** Human Phenotype Ontology (HPO) terms that differed significantly in frequency among 30 infants who died and 282 infants who survived.

HPO term	HPO ID	Alive (%)	Deceased (%)	*P*-value
		*n* = 282	*n* = 30	
Abnormal urinary system	HP:0000079	91 (32.3%)	22 (73.3%)	<0.001
Respiratory failure	HP:0002878	84 (29.8%)	21 (70%)	<0.001
Hypotension	HP:0002615	53 (18.8%)	16 (53.3%)	<0.001
Cardiac arrest	HP:0001695	4 (1.4%)	6 (20%)	<0.001

Terms were extracted from EHRs of 312 infants at time of enrollment for rapid clinical genomic sequencing for diagnosis of genetic diseases^17^.

We examined phenotypic characteristics of all infants who died in San Diego County during the same period, and compared in-hospital deaths with out-of-hospital deaths. Using the SOMI platform^47^, we identified ICD-10 (International Statistical Classification of Diseases and Related Health Problems, 10th Revision) codes in 1,179 infant deaths in San Diego County between 2005 and 2011 (Table 2). Four hundred and thirty-five (37%) infant deaths occurred in a hospital. Among 656 infant deaths, associated with an ICD10 code, the most frequent conditions were gestation <32 weeks (46%), perinatal conditions (45%), congenital malformations and chromosomal anomalies (29%), and sudden infant death syndrome (13%). In-hospital compared to out-of-hospital deaths were enriched for perinatal conditions (63% vs 2.7%; *p*-value < 0.001), congenital malformations (34% vs 5.9%; *p*-value: < 0.001), and circulatory system diseases (3% vs 0.4%; *p*-value: < 0.001). Sudden Infant Death Syndrome was under-represented in-hospital deaths (1.8% vs 9.9%; *p*-value < 0.001).

**Table 2:** ICD10 codes of 1,179 infant deaths in San Diego County between 2005 and 2011.

ICD10 Code	All deaths, *n* (%)	Out-of-hospital deaths, *n* (%)	In-hospital deaths, *n* (%)
Total	1,179	744 (63.1%)	435 (36.9%)
No ICD10 code provided	523	523 (70.3)%	<5 (0%)
Gestation <32 weeks	304 (46%)	304 (40.9%)	n.d.
Gestation 32–36 weeks	62 (9%)	62 (8.3%)	n.d.
Gestation 37–38 weeks	69 (11%)	69 (9.3%)	n.d.
Gestation 39–44 weeks	88 (13%)	88 (11.8%)	n.d.
A -Certain infectious and parasitic diseases	5 (1%)	0 (0%)	5 (1.1%)
C – Neoplasms	5 (1%)	<5 (0.3%)	<5 (0.7%)
D – Blood/immune system diseases	9 (1%)	<5 (0.5%)	5 (1.1%)
E - Endocrine, nutritional and metabolic diseases	11 (2%)	<5 (0.5%)	7 (1.6%)
G - Diseases of nervous system	13 (2%)	5 (0.7%)	8 (1.8%)
I - Diseases of circulatory system	16 (2%)	<5 (0.4%)^*^	13 (3.0%)^*^
J,K,M,N - Diseases of respiratory, digestive, genitourinary and musculoskeletal systems	8 (1%)	<5 (0.3%)	6 (1.4%)
P - Certain conditions originating in the perinatal period	295 (45%)	20 (2.7%)^*^	275 (63%)^*^
Q - Congenital malformations, deformations & chromosomal abnormalities	192 (29%)	44 (5.9%)^*^	148 (34%)^*^
R95 - Sudden infant death syndrome	82 (13%)	74 (9.9%)^*^	8 (1.8%)^*^
R99,Y34 - Ill-defined, unknown, undetermined	6 (1%)	<5 (0.5%)	<5 (0.5%)
V,W,X – Accidents, Assault	14 (2%)	8 (1.1%)	6 (1.4%)

^*^p-value: < 0.001.

In the two RCHSD studies, 81 (26%) of the 312 infants were diagnosed with a genetic disease by rapid genomic sequencing. Of the 30 infants who died, 10 (33%) were diagnosed with 11 genetic diseases (Table 3). The mortality rate, although higher among those with genetic diagnoses, did not differ significantly between infants with or without genetic diseases (12.3%; 10 of 81 versus 8.7%; 20 of 231; *P* = 0.85), respectively. Nor did the average age at time of death differ significantly between infants with or without genetic diseases (104 days versus 89 days, *P* = 0.72). All of the genetic diseases diagnosed in infants who died had previously been associated with infant or childhood mortality, with the exception of Chr 12q21.33q22 del, which has only been described in six children^48–62^. In addition, the symptoms and signs observed in these infants were consistent with causal association of genetic diseases and infant death (Table 3). No genetic disease was recurrent. Of the genetic diseases identified in infants who died, only CHARGE syndrome was diagnosed in at least one of the 282 infants who survived.

**Table 3:** Genetic diseases diagnosed by rapid genomic sequencing in ten of thirty infants who died.

Patient ID	Genetic disease	Affected gene or locus	Age at death (days)	Clinical presentation^a^	Ref.
*244*	Chr 14q31.2q32.2 del syn	14q31.2q32.2 del	25	Metabolic acidosis, RF, sepsis, hypoxic ischemic encephalopathy, coagulopathy, hydrops, pulmonary hypertension	53,54
*247*	CHARGE syn	CHD7	23	CHARGE syndrome	55,56
*302*	Chr 17q12 del syn	17q12 del	4	Skeletal dysplasia, cytopenias, coagulopathy, respiratory failure, hydrocephalus, arthrogryposis	57,58
*311*	Barth syn	TAZ	99	Cardiomyopathy, heart failure, respiratory distress, failure to thrive	59,60
*325*	Campomelic dysplasia w. sex reversal	SOX9	81	Skeletal dysplasia, respiratory distress syndrome, tracheomalacia, hydronephrosis, respiratory failure, hip dysplasia	61
*6012*	Coffin-Siris syn 1	ARID1B	250	Diaphragmatic hernia, pulm hypertension, chronic lung disease, arch hypoplasia, ventricular septal defect, heart failure, atrial flutter, sepsis	62
*6034*	Chr 12q21.33q22 del syn	12q21.33q22 del	29	Prune Belly, dysplastic kidneys, cryptorchidism, pulm hypoplasia, RF, heart failure	63
*6144*	Mitochondrial complex II deficiency	SDHA	196	Cardiomyopathy, chronic lung disease, osteopenia, recurrent infections, leukoencephalopathy, metabolic acidosis	64
*6147*	SIFD; Brugada syn 2	TRNT1; GPD1L	335	Severe microcytic anemia, hepatitis, lethargy, bilateral SN deafness, MA	65,66
*6201*	Mitochondrial complex I deficiency	NDUFV1	2	Metabolic acidosis, intracranial hemorrhage, small for gestational age, respiratory failure, sepsis, pulmonary hypertension, acute kidney injury	67

^a^*Chr* chromosome, *RF* respiratory failure, *del* deletion, *MA* metabolic acidosis, *pulm* pulmonary, *SIFD* sideroblastic anemia with B-cell immunodeficiency, periodic fevers, and developmental delay, *SN* sensorineural, *syn* syndrome, *CHARGE* Coloboma of the eye, heart anomaly, choanal atresia, retardation of mental and somatic development, microphallus, ear abnormalities and/or deafness.

### Systematic literature review

To evaluate whether the findings reported herein were generalizable, we searched the literature for genomic sequencing studies in acutely ill infants in which mortality was recorded. Six additional studies were identified that utilized current genetic disease databases and current variant detection and interpretation methods (Table 4)^25,28,45,63–66^. Three studies had similar experimental designs as herein–diagnostic genomic sequencing was performed in infant inpatients in United States hospitals who had acute diseases of unknown etiology and in whom genetic diseases were suspected^25,28,45^. In two studies, genome sequencing was performed post mortem^63,64^. One study had both *ante* and post mortem genomic sequencing^65^. While all included inpatient and community deaths, the inclusion criteria differed in each study (Table 4). *In toto*, the studies examined 918 infant deaths. The overall contribution of genetic disease to infant mortality was 21% (weighted average, median 31%, range 6 to 86%). Locus heterogeneity (number of affected loci divided by number of infant deaths) was 70% (137 genetic diseases in 195 infant deaths). The most common genetic causes of death were trisomy 21 (9%), spinal muscular atrophy (5%), 22q11 deletion syndrome (4%), trisomy 18 (4%), CHARGE syndrome (3%), and trisomy 13 (2%). It should be noted that most studies excluded infants with known chromosomal anomalies and those who died prior to enrollment, resulting in under-reporting of these. In two of the studies, a further 8% of infant deaths were associated with variants of uncertain significance (VUS) in established disease loci^63,64^. Infant mortality attributable to genetic diseases was not restricted to congenital malformations, nor were all infant deaths associated with congenital malformations attributable to genetic diseases. A literature review identified treatment guidelines for 70% of the genetic diseases diagnosed in the 195 infant deaths. These include case series and case reports, as standard of care practices have yet to be fully established for many exceedingly rare genetic conditions. Furthermore, effective, standard of care treatments were available for 18% (Table 5). Many of these treatments are aimed at surveillance and management of symptoms, although some of them are curative, such as bone marrow transplant in severe cases of autoimmune lymphoproliferative disorder^67–97^. Remaining treatments were supportive or ameliorative. Additionally, genetic evaluation of family members is recommended for all the conditions summarized in Table 5, in particular for autosomal dominant conditions like Alagille Syndrome. Some patients may not be suitable for treatment, and in other cases treatments may not prove effective, in which case palliative and supportive care may be offered. The parents of 31 (35%) of 88 infants who received diagnoses of untreatable genetic diseases before death in our cohort elected palliative care as a result.

**Table 4:** Published and current studies of infant mortality associated with genetic diseases by genomic sequencing.

Ref.	Subjects	Infant deaths abstracted	Time at molecular diagnosis^a^	Age at enrollment	Other inclusion criteria	Percent receiving genomic sequencing	Suspected genetic disease prior to sequencing	Type of sequencing	Enrollment dates	Type of study	Location	Infant deaths associated with genetic disease
68	3989	223	PM	<28 DOL	ICU cohort; Death in hospital or within 1 week of discharge^b^	100%	No	WES	Jan 2015 - Dec 2017	Retrospective	Fudan University, China	6% (13/223)
29	272	58	AM	<100 DOL	ICU cohort; Death by DOL 120^b^	100%	Yes	WES	Dec 2011 - Jan 2017	Retrospective	Houston, TX, USA	52% (30/58)
49	35	14	AM	<4 months	ICU cohort; Death by DOL120^b^	100%	Yes	WGS	Nov 2011 - Oct 2014	Retrospective	Kansas City, MO, USA	86% (12/14)
32	65	10	AM	<4 months	ICU cohort; Infant death^b^	85%	Yes	WGS,WES, panel	Oct 2014 - Jun 2016	Randomized controlled trial	Kansas City, MO, USA	30% (3/10)
69	16	10	PM	<1 year	Infant death without molecular cause at postmortem^b^	100%	No	WGS	Not specified	Postmortem	Brisbane, Queensland, Australia	30% (3/10)
70	573	573	Both	<1 year	In- or out-patient evaluation; Infant death	100%	No	WES, panel	Jan 2011 - Jun 2017	Retrospective	Boston MA, USA	22% (124/573)
Herein, 28	205	14	AM	<4 months	ICU cohort^b^	100%	Yes	WGS,WES	Jun 2017 - Oct 2018	Randomized controlled trial	San Diego, CA, USA	36% (5/14)
Herein	107	16	AM	<1 year	Inpatient cohorts^b^	100%	Yes	WGS	Nov 2015 - Sep 2020	Prospective	San Diego, CA, USA	31% (5/16)
Weighted Average (Median).												21% (31%)

^a^PM: Post-mortem, AM: ante-mortem.

^b^Excluded infants with known chromosomal anomaly.

**Table 5:** 23 examples of genetic diseases associated with infant mortality that have published, effective treatment guidelines.

Locus /Loci	Mendelian inheritance in man or orphanet disease name	Treatment guidance
*JAG1*	Alagille syndrome	66
*FASLG*	Autoimmune lymphoproliferative syndrome	67
*PKHD1*	Autosomal recessive polycystic kidney disease	68
*TAZ*	Barth syndrome	69
*SCN5A, GPD1L*	Brugada syndrome	70, 71
*LMNA*	Cardiomyopathy, dilated, 1A	72, 73
*SLC25A20*	Carnitine-acylcarnitine translocase deficiency	74, 75
*CYBB*	Chronic granulomatous disease, X-linked	76
*RYR1*	Cong. neuromuscular disease with uniform type 1 fiber	77
*CPT2*	CPT II deficiency, lethal neonatal	78
*SCN2A*	Epileptic encephalopathy, early infantile, 11	79–81
*ETFDH*	Glutaric acidemia IIC	82–84
*PRF1, STXBP2*	Hemophagocytic lymphohistiocytosis, familial	85, 86
*IL10RA*	Inflammatory bowel disease 28, early onset, AR	87
*TRMU*	Liver failure, transient infantile	88
*MUT*	Methylmalonic aciduria, mut(0) type	89
*OTC*	Ornithine transcarbamylase deficiency	90
*TCIRG1*	Osteopetrosis, autosomal recessive 1	91
*GAA*	Pompe disease	92
*PDHA1*	Pyruvate dehydrogenase E1-alpha deficiency	93, 94
*SMN1*	Spinal muscular atrophy	95
*CACNA1C*	Timothy Syndrome	96
*TSC2*	Tuberous sclerosis 2	97

## Discussion

Genomic sequencing has greatly increased the number of infants in ICUs who are diagnosed with genetic diseases and holds promise to decrease infant mortality in this setting^15–45^. Little, however, has yet been published with regard to the contribution of genetic diseases to infant mortality using this technology. In six published studies and the cohort reported herein a weighted average of 21% of infant deaths was attributable to single locus genetic diseases^25,28,45,63–65^. However, the range of attribution varied widely between studies (6%-86%), reflecting different experimental designs and inclusion and exclusion criteria. This estimate excluded VUS in disease genes known to be associated with infant mortality. In two studies, the contribution of genetic disease to infant mortality increased 8% upon inclusion of VUS^63,64^. These data also excluded genes of uncertain significance that are known to be loss of function intolerant or associated with perinatal death in animal models. While the observed between-study heterogeneity precludes a precise estimate the contribution of single locus genetic disease to infant mortality, these data suggest that it is considerable. This is important since it informs estimates of potential reduction in infant mortality achievable by genomic medicine and infant suffering through genome-informed palliative care.

All but two of the studies reviewed herein evaluated mortality in infants who had been admitted to a regional or level IV neonatal, pediatric and cardiovascular ICU during the first year of life. Since only ~4% of infants are admitted to regional ICUs, there is concern that findings from these studies may not be generalizable. This concern was offset in part by two observations. Firstly, 37% of infant mortality in San Diego County occurred in hospitals (https://data.chhs.ca.gov/dataset/licensed-bed-classification-and-designations-trends). Secondly, two studies that were not restricted to infants who had been in an ICU had the same weighted average (22%) of infant mortality attributable to genetic diseases as the others. From a public health standpoint, however, it would be of greater value to know the proportion of all infant mortality that is associated with genetic diseases. Many of the most common genetic diseases that contribute to infant mortality, including trisomies 21, 18, and 13 (15% of total deaths) and 22q11 deletions (4% of total deaths), are well-characterized and are often detectable prenatally using maternal cell-free DNA screening. Rapid, accurate identification of these more common genetic conditions in the prenatal period allows for more targeted use of genomic sequencing and more efficient use of this technology for patients with a possible genetic etiology of their illness but without a prenatal diagnosis.

Comparison of causes of in-hospital and out-of-hospital infant mortality in San Diego County revealed significant differences. Mortality associated with perinatal conditions, congenital malformations, and circulatory system diseases was greater in hospitalized infants. Significantly more sudden infant death syndrome occurred in out-of-hospital deaths. Thus the incidence of specific genetic diseases associated with infant deaths in hospital are likely to differ from those that occur out-of-hospital.

More than 14,000 single locus genetic diseases have been described^11,12^, and locus heterogeneity is high among infants diagnosed with genetic diseases. In a cohort of 504 patients diagnosed with genetic diseases by exome sequencing, for example, locus heterogeneity was 72%^98^. A striking finding herein was that locus heterogeneity was similar among infant deaths (70% in 195 infant deaths). Thus, strong negative selection occurs broadly across the genome in human populations. From a practical standpoint, locus heterogeneity implies that effective molecular diagnosis in infants requires comprehensive genomic sequencing, rather than panel tests.

A surprising finding was that published treatment guidelines existed for 70% of infant deaths associated with genetic diseases. Although some patients may not be suitable for treatment even if guidelines exist, it is possible that if substantiated in a larger, unbiased cohort this would imply that there exists a considerable opportunity to decrease infant mortality and morbidity associated with genetic diseases by early neonatal diagnosis. It should be noted that it is now possible to diagnose a genetic disease in 19 h by ultra-rapid whole genome sequencing^17^, albeit such testing is not yet reimbursed by most healthcare payers. There is now a growing literature of infants whose lives have been saved by early diagnosis of genetic diseases and timely implementation of targeted treatments^15–45^.

Another interesting finding herein was that palliative care counseling was provided to 40% of parents of infants who died of genetic diseases. Palliative care counseling was often associated with changes in parental decisions with regard to treatment intensity, such as modified “Code Blue” resuscitation status in the event of cardiorespiratory arrest. If substantiated in a larger, unbiased cohort this would imply that there exists a potential opportunity to decrease suffering in infants with genetic diseases that have fatal prognosis by early diagnosis, and to facilitate a healthy grieving process among parents of deceased infants^99^.

In summary, this study demonstrates that it is possible to identify the contribution of genetic diseases to infant mortality by genomic sequencing. Larger studies using the methods described herein have potential for molecular redefinition of the leading causes of infant death. By analysis of the specific diseases that cause infant mortality it is possible to identify primary and secondary strategies that have the potential to reduce infant mortality and decrease suffering. There was notable heterogeneity in the studies included here, particularly regarding inclusion criteria, geography and initial selection of the cohort. Future studies are therefore warranted that examine large, sequential cohorts of individuals from a single geography over several years in order to more accurately quantify the contribution of genetic disease to overall infant mortality. A larger study could also provide an estimate of the potential impact of genomic medicine on reducing infant mortality. Finally, this study has demonstrated the potential for the SOMI platform to determine whether spatial, environmental, sociodemographic and health-related factors differ among those with and without a known genetic contribution to infant mortality. As with previous population studies of San Diego County, many of the results will have generalizable implications^100–103^.

## Methods

### Cohort studies

We examined infant mortality among 312 children who had received rapid genomic sequencing in two research datasets. The first was NSIGHT2, a prospective, blinded, randomized controlled trial in infants in the neonatal, pediatric and cardiovascular ICUs at Rady Children’s Hospital, San Diego (RCHSD). NSIGHT2 compared the effectiveness and outcomes of rapid whole genome sequencing, rapid whole exome sequencing and ultra-rapid whole genome sequencing (ClinicalTrials.gov NCT03211039, registered July 7, 2017)^24^. NSIGHT2 was approved by the institutional review board (IRB) at RCHSD, was designated non-significant risk by the Food and Drug Administration (FDA) in an Investigational Device Exemption presubmission, and was performed in accordance with the Declaration of Helsinki. Informed consent was obtained from at least one parent or guardian for each infant. The inclusion criteria were age <4 months and time from admission or time from development of a feature suggestive of a genetic condition of <96 h. The clinical inclusion criteria were broad in order to include all acutely ill infants with diseases of unknown etiology, as well as those with presentations highly suspicious for a genetic cause. Infants in whom there was a very low likelihood that a genetic disease diagnosis would change management were excluded. Specific exclusion criteria included infection or sepsis with normal response to therapy, isolated prematurity, and previously confirmed genetic diagnosis that explained their clinical condition. Infants with a confirmed molecular diagnosis of certain common genetic conditions that are detected by prenatal screening were therefore exlcuded. Full inclusion and exclusion criteria have been previously published^24^. Genome interpretation was performed as singleton probands. Infants undiagnosed as singletons were re-analyzed as familial trios.

The second dataset was from a biorepository cohort^17,18,23,30^. Inpatient infants at RCHSD without etiologic diagnoses, in whom a genetic disorder was possible, were enrolled between 11/23/2015 and 9/7/2018. Retrospective comparison of outcomes and healthcare utilization was approved by the IRB at RCHSD and the FDA (ClinicalTrials.gov NCT02917460, registered September 28, 2016). Informed consent was obtained from at least one parent or guardian. Inclusion criteria were symptomatic inpatients less than 1 year of age who were affected by an illness potentially attributable to a genetic disorder. There were no specific exclusion criteria. All patients enrolled underwent whole genome sequencing.

### Clinical, rapid whole genome and exome sequencing, analysis and interpretation

Clinical rapid whole genome sequencing, rapid whole exome sequencing and ultra-rapid whole genome sequencing were performed in laboratories accredited by the College of American Pathologists and certified through the Clinical Laboratory Improvement Amendments^24^. Experts selected a few clinical features representative of each child’s illness from the Electronic Health Record (EHR, Epic) and mapped them to simple genetic diseases with VAAST (Fabric Genomics). Trio EDTA-blood samples were obtained where possible. Genomic DNA was isolated with an EZ1 Advanced XL robot and the EZ1 DSP DNA Blood kit (Qiagen). DNA quality was assessed with the Quant-iT Picogreen dsDNA assay kit (ThermoFisher Scientific) using the Gemini EM Microplate Reader (Molecular Devices). Genomic DNA was fragmented by sonication (Covaris) and bar-coded, paired-end, PCR-free libraries were prepared for rapid whole genome sequencing with TruSeq DNA LT kits (Illumina) or Hyper kits (KAPA Biosystems). Sequencing libraries were analyzed with a Library Quantification Kit (KAPA Biosystems) and High Sensitivity NGS Fragment Analysis Kit (Advanced Analytical), respectively. 2 ×101 nt rapid and ultra-rapid whole genome sequencing was performed to at least 40-fold coverage with Illumina HiSeq 2500 (rapid run mode), HiSeq 4000, or NovaSeq 6000 (S1 or S2 flow cell) instruments, as described^24^.

Sample preparation and sequencing for rapid whole exome sequencing was performed by an external clinical laboratory (GeneDx). Exome enrichment was with the xGen Exome Research Panel v1.0 (Integrated DNA Technologies), and amplification used the Herculase II Fusion polymerase (Agilent)^24^. FASTQ files for rapid whole exome sequencing were transferred to Rady Children’s Institute for Genomic Medicine (RCIGM) for analysis and interpretation^24^.

Rapid whole genome, rapid whole exome and ultra-rapid whole genome sequences were aligned to human genome assembly GRCh37 (hg19), and variants were identified with the DRAGEN Platform (v.3.5, Illumina, San Diego)^24^. Structural variants were identified with Manta and CNVnator (using DNAnexus)^24^. Structural variants were filtered to retain those affecting coding regions of known disease genes and with allele frequencies <2% in the RCIGM database. Nucleotide and structural variants were annotated, analyzed, and interpreted by clinical molecular geneticists using Opal Clinical (Fabric Genomics), according to standard guidelines^24^. Opal annotated variants with respect to pathogenicity, generated a rank-ordered differential diagnosis based on the disease gene algorithm VAAST, a gene burden test, and the algorithm PHEVOR (Phenotype Driven Variant Ontological Re-ranking), which combined the observed Human Phenotype Ontology (HPO) phenotype terms from patients, and re-ranked disease genes based on the phenotypic match and the gene score^24^. Automatically generated, ranked results were manually interpreted through iterative Opal searches. Initially, variants were filtered to retain those with allele frequencies of <1% in the Exome Variant Server, 1000 Genomes Samples, and Exome Aggregation Consortium database^24^. Variants were further filtered for de novo, recessive and dominant inheritance patterns. The evidence supporting a diagnosis was then manually evaluated by comparison with the published literature. Analysis, interpretation and reporting required an average of six hours of expert effort. If genomic sequencing established a provisional diagnosis for which a specific treatment was available to prevent morbidity or mortality, this was immediately conveyed to the clinical team, as described^24^. All causative variants were confirmed by Sanger sequencing or chromosomal microarray, as appropriate. Secondary findings were not reported, but medically actionable incidental findings were reported if families consented to receive this information.

### Infant mortality data collection

Characteristics of infant mortality (up to one year of age) were assessed among the 312 infants by structured EHR queries and manual EHR review. Characteristics included sex, race, ethnicity, clinical presentation, age at time of enrollment for rapid genomic sequencing, molecular diagnosis, age at time of death, “Code Blue” resuscitation status in the event of cardiorespiratory arrest, and whether a palliative care consultation had been performed. HPO terms at time of enrollment were extracted from the Epic EHR using clinical natural language processing as previously described^17^.

### The San Diego study of outcomes in mothers and infants (SOMI)

SOMI is a community-level, spatially referenced research platform with data derived from a complete collection of birth and death records and hospital discharge summaries for all births in San Diego County^47^. Integrated into the platform are spatially referenced data on a wide variety of environmental measures, including census, air and water quality, climate, economic, crime, the built environment, transportation and social system data. The SOMI dataset represents the entire population of ~1 million mothers and their children born between 2005 and 2024. These data can also be linked to banked maternal serum samples from up to 70% of mothers who have received prenatal care in San Diego County, and archived newborn blood spots for virtually all live-born infants in the county. The SOMI dataset can be queried with a unique data visualization platform that incorporates many sources and types of data into one interactive mapping tool. The SOMI platform enables exploration of multi-level data to identify relationships between infant mortality and many pre- and post-natal environmental, genetic and epigenetic factors. The IRBs at the University of California San Diego and San Francisco have approved a waiver of consent for queries of the de-identified SOMI dataset.

### Systematic literature review

A literature review to compare published evidence with findings from the two cohort studies and from data about overall infant deaths occurring in San Diego was conducted according to the MOOSE and PRISMA guidelines.

### Data sources and record identification

We searched PubMed from January 1, 2010, to February 22, 2020 with the terms “infant mortality” and “genetic disease”. There were no language restrictions. There were no study design restrictions.

### Study screening and eligibility

Studies that evaluated the etiologic contribution of single locus genetic diseases to infant mortality using comprehensive genomic methods (genome or exome sequencing with or without chromosomal microarray) were eligible. We limited eligibility to studies of cohorts with a broad range of genetic diseases, rather than one or a few disease types or clinical presentations, and in which the majority of probands were less than 18 years old.

### Inclusion criteria and data extraction

Data extraction was manual. Data was reviewed for completeness and accuracy by at least two expert investigators and disparities were reconciled by consensus. Data extraction was limited to infant deaths which occurred at an age less than 1 year. Inclusion criteria for data abstraction included the use of genome or exome sequencing with or without chromosomal microarray, and cases were considered positive if they had a confirmed diagnosis of a single locus genetic disease detected by sequencing. The primary outcome considered was infant mortality associated with a molecular diagnosis of a genetic disease. Molecular diagnoses were defined as pathogenic or likely pathogenic diplotypes affecting single genes or loci with definitive, strong or moderate associations with infant mortality causation. Variants of uncertain significance and secondary findings were not extracted. Where more than one publication reported results from a cohort, we included the most recent value for diagnostic utility.

Testing was performed clinically in hospital laboratories and reference laboratories, and experimentally in research laboratories. Hospital and reference laboratory clinical tests were defined primarily by the site of testing and, as disclosed in the methods, and, secondarily, by the affiliations of the authors. Clinical testing was defined as testing under fixed protocols that were attested to comply with state or national regulatory guidelines for in vitro diagnostic testing. Experimental research tests were those that explored the utility of novel or bespoke methods of testing or analysis.

### Statistical analysis

For continuous outcomes, such as age at death, *P*-values were determined with a two-tailed *t*-test for independent samples. Counts of outcomes were compared between groups with chi-square. A *z*-test was used to evaluate the association of proportions between populations, using the binomial approximation of the normal distribution. A significance cutoff of 0.05 was used for all comparisons.

### Reporting summary

Further information on research design is available in the Nature Research Reporting Summary linked to this article.

## Supplementary information

Reporting Summary

## Data Availability

All data associated with this study are present in the paper or are available at the Longitudinal Pediatric Data Resource under a data use agreement and subject to the limitations of the informed consent documents for each subject (Accession Number nbs000003.v1.p, https://www.nbstrn.org/research-tools/longitudinal-pediatric-data-resource).
