# Supplementary material for: Measurement of genetic diseases as a cause of mortality in infants receiving whole genome sequencing
Source: NPJ Genom Med. 2020 Nov 2;5:49. doi: 10.1038/s41525-020-00155-8 (PMC7608690; doi:10.1038/s41525-020-00155-8)
Supplement: Supplementary file 1 — Reporting Summary [file 41525_2020_155_MOESM1_ESM.pdf]

nature research

Corresponding author(s): Kingsmore, SF

Last updated by author(s): Aug 11, 2020

Reporting Summary

Nature Research wishes to improve the reproducibility of the work that we publish. This form provides structure for consistency and transparency in reporting. For further information on Nature Research policies, see our Editorial Policies and the Editorial Policy Checklist.

Statistics

For all statistical analyses, confirm that the following items are present in the figure legend, table legend, main text, or Methods section.

n/a	Confirmed	
☐	☒	The exact sample size (*n*) for each experimental group/condition, given as a discrete number and unit of measurement
☐	☒	A statement on whether measurements were taken from distinct samples or whether the same sample was measured repeatedly
☐	☒	The statistical test(s) used AND whether they are one- or two-sided<br>*Only common tests should be described solely by name; describe more complex techniques in the Methods section.*
☐	☒	A description of all covariates tested
☐	☒	A description of any assumptions or corrections, such as tests of normality and adjustment for multiple comparisons
☐	☒	A full description of the statistical parameters including central tendency (e.g. means) or other basic estimates (e.g. regression coefficient) AND variation (e.g. standard deviation) or associated estimates of uncertainty (e.g. confidence intervals)
☒	☐	For null hypothesis testing, the test statistic (e.g. *F*, *t*, *r*) with confidence intervals, effect sizes, degrees of freedom and *P* value noted<br>*Give P values as exact values whenever suitable.*
☒	☐	For Bayesian analysis, information on the choice of priors and Markov chain Monte Carlo settings
☒	☐	For hierarchical and complex designs, identification of the appropriate level for tests and full reporting of outcomes
☐	☒	Estimates of effect sizes (e.g. Cohen's *d*, Pearson's *r*), indicating how they were calculated

Our web collection on statistics for biologists contains articles on many of the points above.

Software and code

Policy information about availability of computer code

Data collection	Clinical natural language processing, described in reference 21
Data analysis	DRAGEN Platform (v.3.5, Illumina, San Diego), VAAST (Fabric Genomics) was used to map phenotype to simple genetic diseases, Manta and CNVnator (using DNAnexus), Opal Clinical (Fabric Genomics), PHEVOR (Phenotype Driven Variant Ontological Re-ranking, Fabric Genomics)

For manuscripts utilizing custom algorithms or software that are central to the research but not yet described in published literature, software must be made available to editors and reviewers. We strongly encourage code deposition in a community repository (e.g. GitHub). See the Nature Research guidelines for submitting code & software for further information.

Data

Policy information about availability of data

All manuscripts must include a data availability statement. This statement should provide the following information, where applicable:

- Accession codes, unique identifiers, or web links for publicly available datasets
- A list of figures that have associated raw data
- A description of any restrictions on data availability

All data associated with this study are present in the paper or are available at the Longitudinal Pediatric Data Resource under a data use agreement and subject to the limitations of the informed consent documents for each subject (Accession Number nbs000003.v1.p, https://www.nbstrn.org/research-tools/longitudinal-pediatric-data-resource).

Field-specific reporting

Please select the one below that is the best fit for your research. If you are not sure, read the appropriate sections before making your selection.

☒ Life sciences ☐ Behavioural & social sciences ☐ Ecological, evolutionary & environmental sciences

For a reference copy of the document with all sections, see nature.com/documents/nr-reporting-summary-flat.pdf

Life sciences study design

All studies must disclose on these points even when the disclosure is negative.

Sample size

This study includes data from 2 cohorts of RCHSD patients, 205 from the NSIGHT2 study and 107 from a biorepository. This cohort was supplemented by abstracted data from the published literature. We specifically examined infant deaths from these cohorts, and included 195 infant deaths in the final analysis. There were no a priori determinations of sample size. Cohort size was maximized by including several different cohorts.

Data exclusions

There are several different populations of infant deaths aggregated in this study. Each individual paper or cohort had its own exclusion criteria, as previously described in those references. Additionally, any infant deaths that occurred after the age of 1 year were excluded from data abstraction. Infant deaths with a previously confirmed genetic diagnosis before sequencing were also excluded from the analysis.

Replication

In order to assess the generalizability of the data from our internal cohort studies, a literature review was carried out and data was abstracted from previously published cohort studies. Due to the nature of the study design, replication was not carried out on any of the cohorts described.

Randomization

Randomization was not directly applicable to this study. This is a cohort study in which we examined whole genome or whole exome sequencing from a cohort of infant deaths taken from internal samples as well as published literature. Randomization was performed for the purposes of the NSIGHT2 trial, from which data is included in the current study. This randomization process has been previously published in reference 28.

Blinding

As above, this is a cohort study examining whole genome or whole exome sequences from a cohort of infant deaths. Blinding was therefore not relevant to this study design.

Reporting for specific materials, systems and methods

We require information from authors about some types of materials, experimental systems and methods used in many studies. Here, indicate whether each material, system or method listed is relevant to your study. If you are not sure if a list item applies to your research, read the appropriate section before selecting a response.

Materials & experimental systems

n/a	Involved in the study	
☒	☐	Antibodies
☒	☐	Eukaryotic cell lines
☒	☐	Palaeontology and archaeology
☒	☐	Animals and other organisms
☐	☒	Human research participants
☐	☒	Clinical data
☒	☐	Dual use research of concern

Methods

n/a	Involved in the study	
☒	☐	ChIP-seq
☒	☐	Flow cytometry
☒	☐	MRI-based neuroimaging

Human research participants

Policy information about studies involving human research participants

Population characteristics

We compared the demographic characteristics of infants who died with those who survived in the two cohorts, and with those of all infant deaths in San Diego County during the same period. Among participants in the two research cohorts, infant mortality did not differ significantly between males (9.5%; 16 of 169) and females (12.3%; 14 of 114; P=0.45). In contrast, in San Diego County from 2015 – 2019, infant mortality was lower among females (0.35%, 282 of 81,612) than males (0.42%, 359 of 85,710, p<0.02)52. Excluding 23 infants of unknown race, mortality in the two studies did not differ significantly between white infants (10.3%; 19 of 185 infants) and those of other races (9.6%; 10 of 104, P=0.86). In contrast, in San Diego County from 2015 – 2019, white infants had a lower mortality (0.34%, 199 of 58,224) than non-white infants (0.41%, 444 of 109,100; p<0.05)52. Excluding 8 infants of unknown ethnicity, mortality did not differ significantly between Hispanic infants (11.7%; 16 of 137 infants) and non-Hispanic infants (8.4%; 14 of 167; P=0.34) in the two studies. In San Diego County from 2015 – 2019, Hispanic infants had a higher mortality rate (0.45%, 304 of 66,928) than non-Hispanic infants (0.34%, 339 of 100,396; P<0.0002)52.

Recruitment

NSIGHT2 Cohort: Patients < 4 months old who were within 96 hours of admission were recruited from RCHSD NICU, PICU and

Recruitment	cardiovascular ICUs. time from admission or time from development of a feature suggestive of a genetic condition of <96 hours. The clinical inclusion criteria were broad so as to include acutely ill infants with diseases of unknown etiology as well as etiologies highly suspicious for a genetic cause. Infants in whom there was a very low likelihood that a genetic disease diagnosis would change management were excluded. Specific exclusion criteria included infection or sepsis with normal response to therapy, isolated prematurity, and previously confirmed genetic diagnosis that explained their clinical condition. Infants with a confirmed molecular diagnosis of certain common genetic conditions that are detected by prenatal screening were therefore exlcuded. Full inclusion and exclusion criteria have been previously published in reference 28.<br>Biorepository Cohort: Inpatient infants without an etiologic diagnosis in whom a genetic disorder was possible were recruited from RCHSD. Inclusion criteria included age < 1 year.
Ethics oversight	NSIGHT2 Cohort: IRB at RCHSD and designated non-significant risk by the FDA, (ClinicalTrials.gov NCT03211039, registered July 7, 2017); Biorepository Cohort: IRB at RCHSD and the FDA (ClinicalTrials.gov NCT02917460, registered September 28, 2016)

Note that full information on the approval of the study protocol must also be provided in the manuscript.

Clinical data

Policy information about clinical studies

All manuscripts should comply with the ICMJE guidelines for publication of clinical research and a completed CONSORT checklist must be included with all submissions.

Clinical trial registration	ClinicalTrials.gov NCT03211039 and NCT02917460
Study protocol	Supplementary Material
Data collection	Samples were collected from infants within the ICUs at RCHSD, as noted above. Biorepository Cohort: 11/23/2015 and 9/7/2018. NSIGHT2 trial: June 2017 - Oct 2018.
Outcomes	Infant (age < 1 year) death, Infant death associated with single locus genetic diseases
